# Mental health outcomes of a peer support programme for prosthesis and orthosis users in Cambodia: An explanatory sequential mixed-methods study

**DOI:** 10.1017/gmh.2026.10276

**Published:** 2026-07-20

**Authors:** Paul Best, Alan Maddock, Thearith Heang, Nil Ean, Sisary Kheng, Nerrolyn Ramstrand

**Affiliations:** 1 Centre for Technological Innovation, Mental Health and Education (TIME), Queenʼs University Belfast, UK; 2 Department of Health Psychology, Royal College of Surgeons in Ireland, Dublin, Ireland; 3 Exceed Worldwide, Phnom Penh, Cambodia; 4 Department of Prosthetics and Orthotics, National Institute of Social Affairs, Phnom Penh, Cambodia; 5 Department of Psychology, Royal University of Phnom Penh, Cambodia; 6 The Center for Trauma Care and Research Organization, Phnom Penh, Cambodia; 7 Department of Rehabilitation, Jönköping University, Jönköping, Sweden

**Keywords:** disability, low-income countries, peer support, mental health

## Abstract

People with physical disabilities in low- and middle-income countries experience elevated rates of psychological distress. This study explored the impact of a peer support intervention (‘friendship group’) for people with disabilities attending prosthetic and orthotic clinics in Cambodia. A total of 462 individuals were screened using the Kessler Psychological Distress Scale (K10), 47 met inclusion criteria (scores 20–29). Of these, 27 enrolled in the intervention, while 20 did not participate. Psychological distress was assessed across four domains of the K10 (nervousness, agitation, fatigue and negative affect). Intervention participants were measured at three time points (2 baseline measures and 1 follow-up), non-participants contributed baseline data only. Quantitative data were analysed using linear mixed-effects models, qualitative data from post-intervention focus groups (*n* = 21) were analysed thematically. Reductions in K10 scores were observed across all domains following the intervention (*p* < 0.001). Qualitative findings highlighted perceived benefits including restored family and community roles, emotional relief and improved self-regulation. Given the pre–post design without a full comparator group, findings should be interpreted cautiously. This exploratory study suggests that peer support may offer a feasible and culturally acceptable route to support people with disabilities in low-resource settings.

## Impact statement

This study shows that a simple, low-cost peer support programme (friendship group) may offer a promising approach to supporting the mental health of people with physical disabilities in Cambodia. Following participation in the eight-week programme, reduction in psychological distress was observed across domains including anxiety, agitation, fatigue and negative mood. These changes were supported by participants’ accounts of feeling more hopeful, more connected and more capable of managing daily challenges.

The friendship group brings together people with shared experiences together in a supportive, stigma-free environment. In a context where mental health continues to be highly stigmatised and access to formal services is limited, participants described valuing the opportunity for open conversation and mutual support. Sharing experiences appeared to reduced feelings of isolation and helped them realise they were not alone in their struggles.

The programme also encouraged small but meaningful actions, such as helping with family tasks or engaging in income-generating activities, this helped rebuild confidence and a sense of purpose. Simple breathing and reflection exercises appeared to support emotional regulation, helping people feel calmer and more in control.

While the study design does not permit firm conclusions about effectiveness, the consistency between qualitative and quantitative findings highlights plausible mechanisms of change, including social connection, renewed purpose and improved emotional regulation. Overall, this exploratory study indicates that peer-led models such as the friendship group may represent a feasible, culturally grounded approach to addressing gaps in mental health support for people with disabilities in low-resource settings.

## Introduction

Physical disability is frequently associated with significant challenges to mental health. Globally, individuals with physical impairments experience higher rates of depression, anxiety and suicidal tendencies compared to the general population (Tough et al., 2017; Akyol Güner and Das Gecim, 2023; Kim and Park, 2024). In low- and middle-income countries (LMICs), these difficulties are often compounded by social and environmental barriers, including stigma, poverty and restricted participation in community life (Bright and Kuper, 2018; Maddock et al., 2023; Mullerpatan et al., 2025). At the same time, access to mental health services in LMIC settings is often limited, leaving many without necessary support (Komu et al., 2025). Identifying contextually relevant interventions that are feasible to promote the psychological well-being of people with disabilities in LMICs is a global health priority and aligns closely with the United Nations’ Sustainable Development Goals’ commitment to inclusive health and well-being (Heymann and Sprague, 2023).

The work presented in this study has been conducted in Cambodia, a lower-middle income country with a troubled history. Cambodia has endured prolonged periods of conflict and violence, beginning with a civil war (1970–1975) and followed by the Khmer Rouge genocide (1975–1979). During this time, it is estimated that approximately one-third of the population died from starvation, disease or execution (Parry and Wilkinson, 2020). The widespread violence and landmine contamination in Cambodia positioned it among those nations with highest proportions of people with disabilities per capita (Gartrell and Hoban, 2013). Current estimates suggest that approximately 9.4% of the Cambodian population lives with a disability, with prevalence increasing markedly with age, reaching 44.2% among individuals over 60 years. This proportion is projected to rise further, potentially doubling by 2050 as the population ages (Kidd et al., 2022).

Cambodia’s violent history has had a major effect on the mental health of its people. During the Khmer Rouge period, all mental health services in the country were destroyed, leaving no mental health professionals to service the population (Parry and Wilkinson, 2020). A process of rebuilding mental health services began in 1992 but, as of 2019, there were only 60 educated psychiatrists in the country (1 per 260,000 people), compared to 1 per 9,300 people in the UK (Parry and Wilkinson, 2020). While the global prevalence of anxiety disorders and depression is estimated at 4% and 5.7%, respectively (Javaid et al., 2023; World Health Organization, 2025), rates among the general Cambodian population have been reported at 27% and 17% (Seponski et al., 2019).

People with physical disabilities experience mental health issues at rates that are much higher than those without disabilities (Cree et al., 2020; Koenig et al., 2024), likely due to greater levels of poverty, limited health care access and social marginalisation. In Cambodia, individuals with mobility impairments requiring prosthetic and orthotic devices represent a particularly vulnerable subgroup, often facing compounded physical, social and economic challenges (Ramstrand et al., 2021). In a recent study of people accessing prosthetic and orthotic services in Cambodia, rates of psychological distress were reported at 21%, while probable post-traumatic stress disorder (PTSD) was observed in 26% of clients (Maddock et al., 2026).

In contexts such as Cambodia, there is an urgent need to identify evidence-based interventions to support the psychosocial well-being of people with disabilities (Maddock et al., 2021). Peer support interventions, in which individuals with lived experience provide emotional and practical support to one another, have gained increasing recognition as a valuable, recovery-oriented approach. Rather than acting solely as a substitute for formal services, peer support offers a complementary model grounded in shared experience, mutual understanding and social connection. Although examples from Cambodia remain limited, recent work by Best et al. (2024) developed and preliminarily tested an eight-week peer support programme (friendship group), offered in both online and face-to-face formats. The study demonstrated that the friendship group model was feasible, culturally acceptable and offered promising reductions in overall psychological distress, PTSD symptoms and worry (Best et al., 2024). However, this earlier work focused primarily on intervention development and delivery format, leaving important questions regarding its application within routine care and its effects on different dimensions of distress.

This study aimed to examine psychological distress and its underlying structure among people with physical disabilities accessing prosthetic and orthotic services in Cambodia, and to evaluate changes in psychological distress following participation in an eight-week friendship group intervention. A secondary aim was to explore participants’ perspectives on the mental health benefits of the friendship group intervention through focus group interviews.

## Methods

### Study design

An explanatory sequential mixed-methods design (Creswell and Piano Clark, 2018) was used to explore changes in mental health outcomes following participation in an eight-week long friendship group intervention. The quantitative phase was conducted first using a pre-test, post-test design to assess for changes in domains of the Kessler Psychological Distress Scale (K10), defined by Brooks et al. (2006). Upon conclusion of the intervention, participants were invited to take part in focus groups to discuss their individual and collective experiences, providing qualitative insight into the mechanisms and contextual factors underlying observed changes.

### Study population and recruitment

This study forms part of a larger mental health screening project involving 462 people with physical disabilities, attending prosthetic and orthotic clinics in two rural locations (Kampong Som and Kampong Chhnang) and one urban location (Phnom Penh) in Cambodia. Data were collected between March 2023 and June 2024. To participate in the mental health screening, clients were required to be over the age of 18, have one or more physical disabilities, be attending one of the three participating clinics for provision or maintenance of any type of prosthetic and/or orthotic device and be able to understand the aims of the research project. Clients who had previously participated in pilot projects conducted by this research team (Maddock et al., 2023; Best et al., 2024) were excluded from this round of mental health screening.

The sample of individuals recruited for the present study consisted of individuals identified in the initial screening process as scoring between 20 and 29 on the K10, a range indicative of mild to moderate psychological distress (Kessler et al., 2003). All individuals within this score range were invited to participate in both the study and an eight-week friendship group designed to support their psychosocial well-being. Of those invited, some individuals chose to participate in the friendship group, while others did not participate in the intervention and contributed baseline (pre-intervention) data only.

Individuals scoring 30 or above on the K10 were excluded, as their scores were indicative of severe psychological distress (Kessler et al., 2003). With their permission, people with scores over 30 were referred to receive treatment from a trained clinical psychologist.

Qualitative data were collected following the completion of the intervention; participants who had taken part in the friendship group were invited to participate in focus group discussions after completing the post-intervention assessment. This study was reviewed and approved by the National Ethics Committee for Health Research in Cambodia (#207 NEHCR).

### Friendship group intervention

The friendship group intervention has been described in detail in a previous publication (Best et al., 2024). The intervention was conducted in person for 8 weeks and included behavioural activation, structured peer discussion and simple psychoeducational exercises (Best et al., 2024; Maddock et al., 2025).

### Data collection

#### Quantitative phase

Participants were requested to complete a survey on three occasions:

1. Upon attending one of the three participating prosthetic and orthotic clinics (pre-test 1).
2. Eight weeks after the initial clinic visit but prior to participating in the intervention (pre-test 2).
3. Immediately following the final friendship group intervention (post-test).

The survey was designed to capture participants’ demographic and social characteristics as covariates, while assessing non-specific psychological distress, measured using the K10 as the primary outcome. All items were presented in Khmer, the national language of Cambodia, using a digital form created in Qualtrics (Qualtrics, Provo, Ut.). Participants could choose to compete the survey themselves or, in cases where they had low levels of literacy, have research assistants read the questions and assist in inputting their responses.

Demographic and social variables included items related to participants’ age (6 predefined response alternatives), sex (male/female), geographic setting (urban/rural), level of education (5 response alternative), cause of disability (16 response alternatives) and the type of assistive device they required (prosthesis or orthosis). Participants were also requested to complete a PTSD screening tool, the Primary Care PTSD Screen for DSM-5 (PC-PTSD-5), which included an initial question to determine if they had been exposed to any traumatic event in the past and, when this was the case, posed 5 symptom-related items, each scored on a scale of 0 to 5 (Prins et al., 2016). The PC-PTSD-5 has been translated into Khmer and found to be internally consistent (Maddock et al., 2023).

Mental health was assessed using the Khmer version of the K10 (NSW Government Health, 2012). This instrument was developed to assess psychological distress in the general population (Kessler et al., 2003) and consists of ten items measuring symptoms of anxiety and depression experienced in the past 4 weeks. Each item is scored on a 5-point Likert scale ranging from 1 (none of the time) to 5 (all of the time). Total scores range from 10 to 50, with higher scores representing a greater degree of psychological distress. Total scores are typically interpreted as low (10–19), mild (20–24), moderate (25–29) and severe (30–50) (Andrews and Slade, 2001). Translations of the K10 are widely used in many countries, including Cambodia (Sopheab et al., 2020; Maddock et al., 2023). The English version of the instrument has demonstrated acceptable internal consistency, factor structure and reliability (Sampasa-Kanyinga et al., 2018; Wojujutari and Idemudia, 2024) as well as good cross-cultural validity (Ametaj et al., 2024). The Khmer version, which was used on the same population as in the present study, has been reported to have high internal consistency (Chronbach’s *α* 0.95) (Maddock et al., 2023). However, to the authors’ knowledge, other key psychometric properties of the Khmer version, particularly its underlying factor structure, have not yet been evaluated. Establishing the factor structure of the Khmer K10 was considered important to ensure that the construct of psychological distress is being measured consistently and meaningfully within this cultural context.

Studies examining the factor structure of the English version of K10 generally support a unidimensional construct as well as two latent constructs (depression and anxiety) (Brooks et al., 2006; Peixoto et al., 2021). Some who support the two-factor structure suggest that the two factors may be subdivided into second order factors. Brooks et al. suggest that anxiety is represented by a nervous factor and an agitation factor while depression is represented by fatigue factor and a negative affects factor (2006). Hoffman et al. suggest a slightly different factor model whereby nervousness is combined with fatigue (2022).

#### Qualitative phase

Upon completion of the eight-week friendship group intervention, participants (*n* = 21) were invited to take part in focus groups to share their experiences of the group and how they perceived it to impact on their mental health. Two online focus groups were led by Cambodian facilitators (clinical prosthetists/orthotists who had received training in qualitative methods). Each focus group session was audio-taped, transcribed verbatim in their original language and then translated into English by one of the Cambodian facilitators. Careful attention was directed towards preserving the original meaning, nuance and cultural context of the Khmer version. The focus groups were semi-structured in nature with interviewers drawing upon a topic guide to inform the session. Key topics for discussion included perceived impacts and outcomes, experiences of participation, views on group-based support, negative experiences and refinements.

### Data analysis

#### Quantitative phase

Although the Khmer translation of the K10 has been widely used (Sopheab et al., 2020; Maddock et al., 2025), its psychometric properties (factor structure, reliability and validity) have not been formally assessed. To support the interpretation of the outcome measure in this specific cultural and linguistic context, we conducted a confirmatory factor analysis (CFA) as an initial, secondary analysis. K10 data from the initial screening of 462 clients were used. Four models were tested: the most common one-factor model, a two-factor model proposed by Brooks et al. (2006), Hoffmanʼs four-factor model (2022) and Brooks et al.’s four-factor model (2006). Model fit was assessed using multiple fit indices, including the Comparative Fit Index (CFI), Tucker–Lewis Index (TLI), Root Mean Square Error of Approximation (RMSEA) and Standardised Root Mean Square Residual (SRMR).

To examine whether K10 scores were stable over time prior to the intervention, a linear mixed-effects model (LMM) was used to assess changes between pre-test 1 and pre-test 2, with random intercepts for participants to account for repeated measures. Changes in K10 constructs over time were examined using LMMs, with each construct of the K10 analysed in a separate model. Time (pre-test 1, pre-test 2 and post-test) was modelled as a fixed effect to assess whether scores differed across measurement occasions. Potential confounders included; age (dichotomised as <55 vs. ≥55), sex (male/female), geographical location (urban/rural), disability cause (landmine vs. other), exposure to trauma (yes/no) and education (entered as continuous variable). Participant identity was included as a random intercept to account for repeated measurements within individuals. Model assumptions were assessed via residual plots and Q-Q plots to ensure normality and homoscedasticity. As non-participants did not contribute follow-up data, the study does not include a full comparator group for evaluating intervention effects. Post-hoc pairwise comparisons with Tukey adjustment were conducted to identify time points with significant differences. All analyses were performed in RStudio 2024.04.2, Build 764 (Posit team, Boston, MA.) and the lme4, lmerTest and Emmeans packages.

#### Qualitative phase

Analysis of focus group data adopted a reflexive thematic analysis approach by Braun and Clarke (2021) drawing on their six-phase process to analyse the impact of the friendship group intervention on participants with physical disabilities. Braun and Clarke’s (2021) reflexive model sees the researcher as active in making sense of their data and prioritises an iterative and flexible approach. Initial coding was conducted manually, drawing on both semantic content and underlying latent meanings. Codes were reviewed collaboratively by the research team and refined through iterative discussion, with similar codes grouped inductively into broader thematic categories. Reflexivity was sustained throughout with researchers acknowledging the sociocultural location of the research team and translation as a mediator of meaning. As such, themes were not constructed as simply descriptive rubrics, but as engaging interpretations that sought to illuminate both personal dimensions and the collective social significance (Braun and Clarke, 2021).

A second investigator (NR) reviewed the coded transcripts and any discrepancies in coding were discussed and reconciled with a third member of the research team (AM) to maintain trustworthiness and credibility. We refined the theme structure iteratively, repeatedly checking against the transcripts to ensure each theme was coherent, distinct and firmly grounded in the data. The analysis made a point of focusing on participants’ language and experience of psychosocial shifts and locating them against the broader cultural and contextual landscape of post-conflict Cambodia. Final themes were reviewed by native Cambodian members of the research team to ensure cultural appropriateness of interpretation.

## Results

### Quantitative phase

A total of 462 clients were screened when they attended one of the three prosthetic and orthotic clinics. The total K10 score was used as a screening tool to identify individuals eligible for inclusion in the present study. Of the 462 clients screened, 91(19.7%) recorded total K10 scores between 20 and 29 while 12 (11%) recorded scores of 30 and above. Forty-seven met the inclusion criteria for this study. Of these 47 individuals, 27 participated in the friendship group intervention, while 20 did not participate in the intervention and contributed baseline (pre-intervention) data only. Reasons for not participating were; did not attend booked session (*n* = 3), too busy (*n* = 4), could not be contacted (*n* = 3), ill health (*n* = 2), changed their mind (*n* = 1), moved to another province (*n* = 1), unknown (10). Details of all participants, including a breakdown of those who did and did not participate in friendship groups are presented as Table 1.

**Table 1.** Summary of participant characteristics Table 1. long description.

Variable	Total, *N* (%)	Pre-test data only (*n* = 20)	Pre-test + Post-test data (*n* = 27)
Age			
18–24	0	0	0
25–34	0	0	0
35–44	11 (23.4)	6 (30.0)	5 (18.5)
45–54	9 (19.1)	4 (20.0)	5(18.5)
55–63	22 (46.8)	8 (40.0)	14 (51.9)
64+	5 (10.6)	2 (10.0)	3 (11.1)
Sex			
Male	40 (85.1)	17 (85)	23 (85.2)
Female	7(14.9)	3 (15.0)	4 (14.8)
Marital status			
Single	2 (4.2)	0	2 (7.4)
Married	41 (87.2)	18 (90)	23 (85.2)
Divorced	1 (2.1)	0	1 (3.7)
Widowed	3 (6.3)	2 (10)	1 (3.7)
Geographic setting			
Urban	14 (29.7)	5 (20)	9 (33.3)
Rural	33 (70.2)	15 (75)	18 (66.7)
Average income/month			
No income	3 (6.3)	1 (5)	2 (7.4)
<150 USD	2 (4.2)	1 (5)	1 (3.7)
150–250 USD	9 (19.1)	5 (25)	4 (14.8)
250–400 USD	5 (10.6)	3 (15)	2 (7.4)
400 + USD	0	0	0
Do not wish to disclose	28 (59.6)	10 (50)	18(66.7)
Education			
None	12 (35.5)	6 (30)	6 (22.2)
Primary	22 (46.8)	10 (50)	12 (44.4)
Secondary	9 (19.1)	4 (20)	5 (18.5)
High school	4 (8.5)	0	4 (14.8)
College/university	0	0	0
Cause of disability			
Trauma (landmine)	21 (44.7)	8 (40)	13 (48.1)
Trauma (other)	19 (40.4)	8 (40)	11 (40.7)
Infection	0	0	0
Vascular disease	3 (6.4)	2 (10)	1 (3.7)
Congenital	1 (2.3)	1 (5)	0
Cancer	0	0	0
Other	3 (6.4)	1 (5)	2 (7.4)
Type of assistive device			
Prosthesis	40 (85.1)	16 (80)	24 (88.9)
Orthosis	7 (14.9)	4 (20)	3 (11.1)
PC-PTSD-5 (at pre 1)			
Report having experienced a traumatic event	21	12	9
PTSD score below threshold (<3)	10	8	2
PTSD score above threshold (≥3)	11	5	6

*Note:* Measured at pre-test 1 unless stated.

### Confirmatory factor analysis (CFA)

To support interpretation of the K10 in the Cambodian context, a CFA was conducted to evaluate the underlying factor structure of the Khmer version and inform the selection of outcome variables for subsequent analyses. CFA was performed using data from all 462 clients who were screened between March 2023 and June 2024. Results of one-factor, two-factor and four-factor models are presented as Supplementary Table S1.

The one-factor model, assuming that all K10 items load onto a single latent factor, showed mixed results (*χ* ^2^(35) = 421.18, *p* < 0.001; CFI = 0.83; TLI = 0.79; RMSEA = 0.154; SRMR = 0.068). All loadings were significant (0.595–0.838), indicating moderate to strong associations with the latent factor. Overall, the model captures the general structure of the items, but some discrepancies exist between the predicted covariance matrix and the observed matrix.

The two-factor model grouped four items into F1 and six items into F2 (Brooks et al., 2006). This model showed moderate fit: *χ* ^2^(34) = 346.71, *p* < 0.001; CFI = 0.87; TLI = 0.83; RMSEA = 0.141; SRMR = 0.055. All factor loadings were significant (0.61–0.84) and reliability was acceptable for both factors (F1 = 0.81, F2 = 0.84). The two factors were strongly correlated (*r* = 0.86), which may suggest overlap between the constructs.

Both four-factor CFA models showed significant factor loadings and generally acceptable reliability. The Hoffman model (Hoffman et al., 2022) had extremely high correlation between Factor 1 (items 7 and 9) and Factor 4 (items 4, 8 and 10) (*r* > 1), suggesting that these items are behaving as a single factor. The Brooks et al. model (2006) improved fit slightly (CFI 0.924, TLI 0.882, RMSEA 0.114, SRMR 0.038) and reduced extreme correlations, although Factor 3 (fatigue – items 1 and 8) and Factor 4 (negative affect – items 4, 7, 9 and 10) were highly correlated. Standardised loadings across both models ranged from moderate to strong (0.58–0.82), and reliability coefficients were mostly satisfactory. Both models produced large modification indices, suggesting potential cross-loadings due to overlapping constructs. Based on these findings, the four-factor model proposed by Brooks et al. (2006) was selected to derive domain-level scores, which were used in all subsequent inferential analyses.

#### Findings from quantitative phase

**Figure 1.**
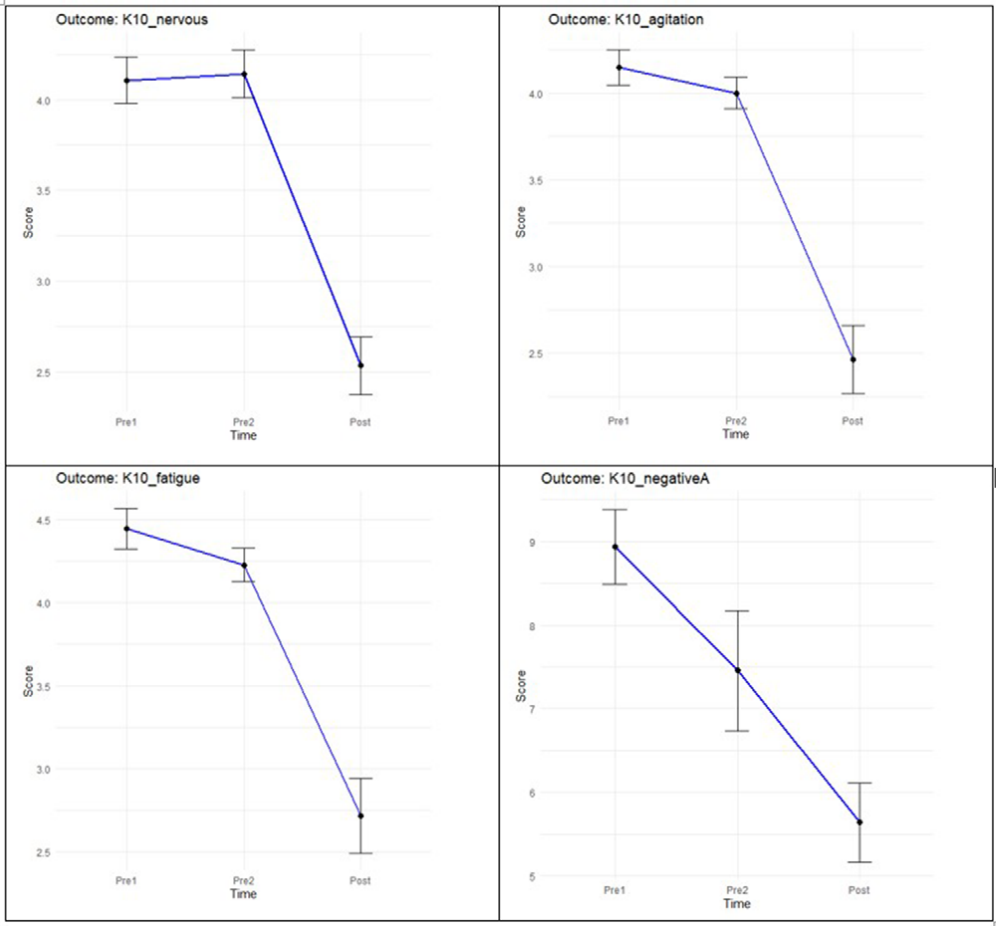
Mean scores (standard error) for K10 constructs at 1/baseline (pre 1), 2/after an eight-week hiatus (pre 2) and 3/after the intervention (post). Figure 1. long description.

Figure 1 presents the unadjusted mean scores for each K10 domain in the four-factor solution. Error bars represent the standard error of the mean, showing that the change after the intervention is larger than the variability within each time point. To examine the stability of psychological distress prior to the intervention (pre-test 1 vs. pre-test 2), LMMs were conducted across each K10 domain. No significant changes were observed in any domain (nervous *p* = 0.84; agitation *p* = 0.42; fatigue *p* = 0.56; and negative affect *p* = 0.06), indicating stability in symptom levels during the pre-intervention period. In addition, no differences were observed between individuals who later participated in the intervention and those who did not across any domain at either pre-intervention time point (nervous *p* = 0.57; agitation *p* = 0.48; fatigue *p* = 0.92; and negative affect *p* = 0.08).

### K10 domains

Across all K10 domains (nervous, agitation, fatigue and negative affect), LMMs consistently showed a significant main effect of the intervention, with higher symptom scores at both pre-test 1 and pre-test 2 compared with post-test (all *p* < 0.01; see Supplementary Tables S2–S5). Post-hoc Tukey-adjusted comparisons confirmed that scores were significantly lower at post-test relative to both pre-test time points across all domains (all *p* < 0.0001), while no significant differences were observed between pre-test 1 and pre-test 2 when accounting for within-participant correlation and adjusting for covariates.

Covariate effects were largely non-significant across domains. However, trauma exposure was consistently associated with higher scores in the agitation, fatigue and negative affect domains (all *p* < 0.05), but not in the nervous domain. Additionally, sex showed a trend towards significance in the negative affect domain, with males reporting higher scores (*p* = 0.053). No other demographic or clinical variables were significantly associated with domain scores.

Random intercept variance was negligible or small across models, indicating limited between-participant variability beyond the fixed effects. Detailed model estimates and fit indices are provided in Supplementary Tables S2–S5.

### Findings from qualitative phase

**Figure 2.**
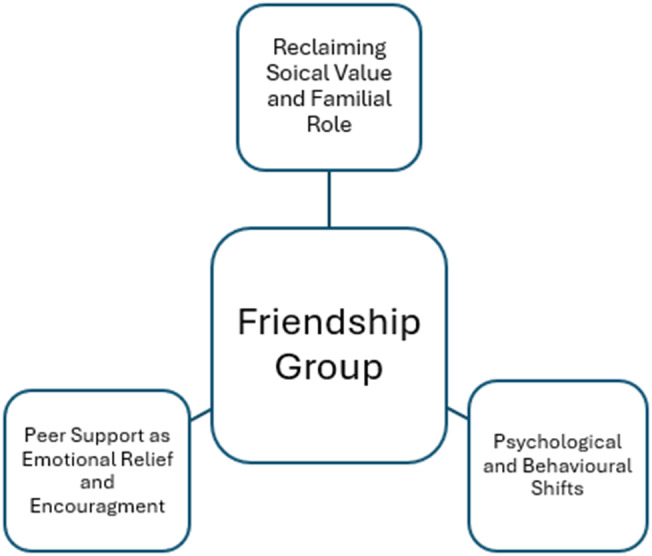
Overview of core themes.

Post-intervention, focus group interviews (*n* = 2) were conducted to seek participant reflections and experiences with a total of 21 participants. This included adults with various physical disabilities resulting from amputation (*n* = 18), stroke (*n* = 1) or polio (*n* = 2). Most people with amputations had sustained their disability from a landmine explosion or road traffic accident. Not all participants knew their exact age, however, based on their own estimations, 8 were aged between 30 and 40, 8 between 50 and 60 and 5 between 60 and 70. Figure 2 presents core themes that emerged from the analysis.

### Theme 1: Reclaiming social value and familial role

One of the most salient themes reported by participants post-friendship group intervention was a renewed sense of purpose and contribution. Participants who had previously felt sidelined by their disability described reclaiming everyday roles within their families and communities. This included supporting household tasks, taking on small-scale income-generating activities and regaining a sense of dignity through productive engagement. For example, one participant described how they had re-engaged with everyday family responsibilities:

Even though I am a person with disabilities, I am able to do small tasks for my family like cutting firewood and taking good care of my buffaloes… I look after my grandchildren and have a small chicken farm. – P1

For others, this shift represented a profound transition from despair to renewed participation and hope:

Before, I just wanted to kill myself by taking rat poison. I felt so sad when I saw others could do something to earn money but I was not able to do so after becoming an amputee… after [the friendship group] I began to grow bananas, coconuts, and crops and look after cows. – P8

This theme also extended to longer-term aspirations. A few participants expressed the desire to start microbusinesses or re-engage with vocational skills using the knowledge they had picked up from others during the groups. For instance, one participant expressed an ambition to pursue service-oriented work, stating:

My future objective is to run a salon where I could learn hair styling, hair washing, nail smoothing, and facial cosmetics. – P2

Others emphasised more practical, livelihood-oriented skills acquired through peer learning, such as small-scale farming and fishing practices, which were seen as achievable and contextually relevant opportunities. As one participant noted:

Laying a fishing net and raising chickens are skills I learned from the group members. – P9

Beyond practical activities, participants described changes in terms of how they altered the way they saw themselves and how they were perceived by others. For some, participating in family and community life was associated with a renewed sense of social worth and recognition. One participant remarked that neighbours had begun to use his efforts as a positive example for their children:

My neighbors educated their children. They said, look at him, even though he is an amputee, he is putting a lot of effort into doing his business. They admired that. – P6

Others reflected on the importance of demonstrating capability and contribution despite their disability, suggesting that this could challenge negative stereotypes and reduce discrimination.

We should be committed and diligent in doing something that we are able to do. When others saw that they would like us. That would be a means to reduce discrimination and they would not look down on us because we are able to do our jobs. – P4

These accounts suggest that reclaiming meaningful social roles was not solely associated with economic contribution, but also with dignity, social recognition and a renewed sense of personal value within the wider community.

Importantly, these shifts did not appear framed as dramatic economic transformations but rather as psychologically meaningful returns to agency, framed within the participants’ sociocultural context and capacities. The ability to ‘be useful again’ appeared linked to self-worth and family identity and appeared to resonate with the overall decline in psychological distress as seen in quantitative data.

### Theme 2: Peer support as emotional relief and encouragement

Many participants spoke of the emotional burden they had carried silently for years, with little opportunity to speak about their experiences or feelings. The friendship group appeared to provide a space in which their struggles were heard, affirmed and normalised. For example,

The [friendship group] gave us a good opportunity to exchange ideas and experiences. We tried to understand the hardships participants shared, and we gave advice to those in difficult situations to motivate them. – P4

For some, the experience of speaking within a group of peers with similar impairments allowed a form of catharsis and belonging.

We had a chance to share our daily experiences with each other. We opened our hearts to learn, to listen, and to accept. Especially, we made friends. – P3

Others noted that the facilitator’s role in guiding reflection made it feel like a structured space rather than a casual conversation.

I gained experiences from brothers and sisters, and especially from the master who taught me how to calm my mind and emotions. This made me feel refreshed. – P7

This sense of collective recovery was especially meaningful in a cultural context where disability is often stigmatised, and emotional openness can be difficult to express. Reductions in nervousness and agitation as seen in K10 appear to correspond with participants’ descriptions of the groups as emotionally safe spaces where they could share experiences, feel understood and normalise their struggles. This peer connection appears to have lessened anxiety and emotional tension.

### Theme 3: Psychological and behavioural shifts

Although the friendship group was not overtly psychotherapeutic, many participants reported internal psychological changes and altered behaviours when around others. Some noted improvements in their ability to regulate emotions such as anger or sadness, often linked to the breathing and reflection exercises introduced in the sessions.

I was taught that we should make ourselves free from cruel, unmerciful attitudes because it does not produce any good outcome. – P10

An interesting sub-theme was the shift in how participants responded to stigma and discrimination. Whereas many initially described internalised shame or avoidance, post-intervention they described standing up for themselves or reasserting their right to be treated with respect.

I told them, when they were riding motorbikes at intersections: if you drive fast and others drive fast, you will collide. I have seen many people die from this kind of driving. So I told them to stop insulting people. Now, people with disabilities have the right to take legal action. I spoke to them like that. Now it is okay. Every day at 4 a.m., they come to my house to drink coffee. We sit together like before, and our friendship has returned to normal. There are no more quarrels or insults. – P7

In some cases, these changes appeared to ripple outwards, influencing the attitudes of neighbours and family members. Others described learning to ‘let go’ of frustration, even without fully confronting others, suggesting a growth in psychological flexibility.

My neighbors admired me when they saw that I stopped drinking alcohol since I joined the friendship group. – P4

This increased ability to regulate emotions and respond differently may help to interpret the observed reduction in negative affect scores captured in K10 data.

### Integration of qualitative and quantitative findings

Overall, there was strong convergence between reductions in K10 domain scores and participants’ accounts of improved emotional well-being, social connection and functional engagement (see Table 2). For example, decreases in nervousness and agitation scores aligned with participants’ descriptions of the friendship group as a safe space for emotional expression and mutual support. Similarly, reductions in negative affect corresponded with narratives of increased confidence improved emotional regulation and a renewed sense of self-worth.

**Table 2.** Integration of quantitative K10 domain changes and qualitative themes Table 2. long description.

Quantitative domain (K10)	Quantitative result	Qualitative theme(s)	Illustrative interpretation
Nervousness	Significant reduction post-intervention	Theme 2: Peer support as emotional relief and encouragement	Sharing experiences and receiving support may have contributed to reduced anxiety and emotional tension.
Agitation	Significant reduction post-intervention	Theme 2: Peer support as emotional relief and encouragement; Theme 3: Psychological and behavioural shifts	Participants described feeling calmer and better able to manage emotional reactions.
Negative affect	Significant reduction post-intervention	Theme 3: Psychological and behavioural shifts	Improved emotional regulation and fewer interpersonal difficulties may help explain reductions in negative affect.
Fatigue	Significant reduction post-intervention	Theme 1: Reclaiming social value and familial Role	Re-engagement in meaningful family and community roles appeared linked to greater purpose and motivation.
Not captured in K10	–	Theme 1, 2 and 3 (cross-cutting)	Social connectedness, hope, empowerment and relational belonging were central to participants’ experiences but not directly measured

Findings relating to fatigue and functional engagement were complemented by participants’ reports of re-engagement in daily activities and livelihood tasks, suggesting that psychological improvements were reflected in behavioural changes.

At the same time, the qualitative data expanded on aspects not captured by the K10, particularly social connectedness, hope and empowerment, highlighting the added value of the qualitative component in understanding recovery processes beyond distress reduction.

## Discussion

This study explored changes in psychological distress following participation in an eight-week friendship group intervention targeting adults with physical disabilities in Cambodia. Quantitative analysis showed reductions across all domains of the K10 (nervousness, agitation, fatigue and negative affect) following the friendship group intervention. Focus group interview data provided depth and explanation to these trends, revealing the interpersonal, emotional and cultural mechanisms that may help to interpret the changes observed in the quantitative phase.

Baseline K10 scores (avg total score = 21.8) were comparable to those reported in our earlier work (avg total score 21.7), while post-intervention scores were somewhat lower in the present study, with an average score of 12.05 compared to an average of 16.5 in our previous work (Best et al., 2024). This difference may reflect increased facilitator experience, as the same individuals delivered the intervention in both studies, and may have refined their delivery over time. In addition, the earlier study was conducted during the COVID-19 pandemic, a period associated with elevated psychological distress, which may have influenced outcomes.

When considered alongside the qualitative findings, the reductions in psychological distress observed in the quantitative data appeared to be accompanied by broader changes in social value, emotional well-being and interpersonal connectedness. Participants consistently described the friendship groups as providing a supportive environment in which they could share experiences, receive encouragement and feel understood by others facing similar challenges. Qualitative accounts also pointed to renewed engagement in family and community roles, enhanced self-worth and greater confidence in managing emotional difficulties. Collectively, these findings suggest that the impact of the intervention may extend beyond reductions in distress and reflect wider processes of recovery and social reintegration.

### Changes across K10 domains

When interpreting changes across K10 domains, differences in scale should be considered. The negative affect domain had a maximum score (20) compared to the other domains (10) (Brooks et al., 2006), yet similar patterns of improvement were observed across all domains. This suggests a broad and relatively balanced reduction in psychological distress following the intervention.

The observed changes are consistent with the theoretical underpinnings of behavioural activation, which encourages re-engagement in meaningful activities (Martell et al. 2021). Improvements in fatigue and mood may therefore reflect increased participation in daily tasks and a renewed sense of purpose rather than purely symptom reduction. Similarly, reductions in negative affect may indicate improvements in emotional regulation rather than temporary mood changes. Participantsʼ qualitative accounts of renewed family and community involvement, enhanced self-worth and greater confidence in managing emotional difficulties offer further insight into the processes through which these improvements may have occurred.

### Influence of trauma and contextual factors

Among the covariates examined, previous exposure to trauma was the only variable associated with higher levels of distress across several domains. This aligns with broader literature related to the impact of trauma, which highlights that individuals with a history of traumatic experience have higher levels of psychological distress (Magruder et al., 2017). Other covariates, including age, sex, geographic location and disability type were not significantly associated with outcomes, suggesting similar patterns of change across demographic subgroups, although causal inferences cannot be drawn.

These findings should be interpreted within the broader socio-economic context. Participants often experienced ongoing challenges including poverty, stigma and social exclusion, which may limit the extent or sustainability of improvements. Similar constraints have been reported in other low-resource and post-conflict settings (Barbui et al., 2020).

### Potential mechanisms of change

Drawing on the integrated quantitative and qualitative findings, the intervention may operate through several interrelated mechanisms. First, social connection may reduce isolation and normalise emotional distress through shared experiences. Second, role restoration may enable participants to re-engage in family and community life, contributing to improved self-worth. Third, elements of emotional regulation, supported through structured discussion and reflection, may enhance participants’ ability to manage distress. These mechanisms are consistent with community-based psychosocial approaches and highlight the importance of relational and contextual factors in mental health recovery (Hobfoll et al., 2007).

### Implications for practice and research

The friendship group model may represent a culturally appropriate and scalable approach in settings where formal mental health services are limited. By emphasising peer interaction, shared experience and practical problem-solving, the intervention aligns with local social structures and may offer a sustainable alternative to more resource-intensive models of care.

Future research should build on these findings by testing the intervention in larger samples and more diverse settings, while incorporating outcome measures that capture broader recovery-oriented constructs alongside psychological distress.

### Strengths and limitations

A key strength of this study lies in its sequential design, which enabled potential changes to be interpreted alongside participant testimony, providing both quantitative and experiential insight. However, several limitations should be noted.

The modest sample size and potential selection bias among participants who attended the groups, who may have been more motivated or socially engaged than non-attenders, limits generalisability. Economic insecurity is also a constraint with participants describing competing demands related to obtaining food or travelling to attend sessions.

The absence of a control group and lack of follow-up among non-participants is a further limitation. Although a group of eligible non-participants contributed baseline data, they were not followed post-intervention and were not randomly assigned. As such, observed improvements cannot be definitively attributed to the intervention rather than other contextual or time-related factors. This study should therefore be considered exploratory rather that confirmatory.

While the intervention was grounded in peer interaction and participant experience, individuals with lived experience were not involved in the formal planning, design or implementation of the study. This may have influenced the extent to which study procedures and outcome measures fully reflected priorities of people with disabilities in the Cambodian context.

Measurement-related limitations should also be considered. While the K10 is a well-established measure of psychological distress, it does not capture recovery-oriented constructs such as social connectedness, hope and empowerment, which are particularly relevant to peer-based interventions (Leamy et al., 2011). Although these dimensions were explored in depth in the qualitative component, their absence in the quantitative assessment limits our ability to examine them systematically or to triangulate findings across methods. In addition, while initial CFA supported the use of the Khmer K10, a comprehensive psychometric evaluation was beyond the scope of this study, limiting the strength of inferences about change over time.

Variation in participant engagement also represents a limitation. Some individuals expressed their reflections in detailed and transformative ways, while others contributed minimally or framed their experience in more practical or surface-level terms. This may reflect differences in readiness, confidence or capacity to engage. Combined with external factors such as economic hardship and stigma, this suggests that the impact of the intervention may have been uneven.

Finally, the translation of qualitative data from Khmer to English may have resulted in some loss of nuance in tone, meaning and cultural context. The presented quotations should therefore be interpreted as approximations of participants’ original expressions.

Future research should test the model with larger and more diverse samples, explore hybrid or digital delivery formats to improve accessibility and consider adaptations for individuals with trauma histories, including additional psychological or socio-economic support components.

## Conclusions

The findings of this mixed-methods study provide preliminary evidence suggesting that the peer-led friendship group may support improvements in the mental well-being of people with physical disabilities in Cambodia. Over an eight-week period, the participants in the peer support programme showed reductions in psychological distress across all measured domains, with post-intervention K10 scores substantially lower than at baseline. Focus group data revealed improvements wherein participants spoke of reclaiming their sense of purpose, finding emotional relief through shared experience and undergoing positive shifts in outlook and behaviour. Taken together, results suggest that potential mechanisms of change in the friendship group may include social connections, mutual support and renewed personal agency. Participants who once felt isolated and hopeless reported feeling valued, understood and better equipped to cope with life’s challenges, and these changes were mirrored in the observed declines in anxiety, agitation and depressive symptoms. This study thus highlights how a low-cost, community-based peer support intervention can bridge gaps in mental health provision for a marginalised population. In a context like Cambodia, where formal psychological services are scarce, empowering persons with disabilities to support one another offers a culturally resonant and sustainable model for mental well-being. While this was not a randomised trial, the consistency of the outcomes and the explanatory insights from participants provide converging evidence consistent with potential benefit, although causal conclusions cannot be drawn given the study design. In future, scaling up such peer-led programme and evaluating them over longer periods will be an important next step.

## Supporting information

10.1017/gmh.2026.10276.sm001 Best et al. supplementary material Best et al. supplementary material

## Data Availability

The datasets used and analysed during the current study are available from the corresponding author on reasonable request.

## Supplementary material

The supplementary material for this article can be found at http://doi.org/10.1017/gmh.2026.10276.

## Long descriptions

### Table 1. Long description

The table consists of four columns and multiple rows categorized by demographic and clinical variables.

* Age: The largest group is 55 to 63 years old at 46.8 percent (22 participants), followed by 35 to 44 at 23.4 percent (11 participants). No participants were under 35.

* Sex: 85.1 percent (40) are Male and 14.9 percent (7) are Female.

* Marital status: 87.2 percent (41) are Married, with small numbers for Single, Divorced, or Widowed.

* Geographic setting: 70.2 percent (33) live in Rural areas, while 29.7 percent (14) are Urban.

* Average income per month: 59.6 percent (28) did not wish to disclose. 19.1 percent (9) earn 150 to 250 U S D. No participants earn over 400 U S D.

* Education: 46.8 percent (22) have Primary education, 35.5 percent (12) have None, and 19.1 percent (9) have Secondary education.

* Cause of disability: Trauma from landmines accounts for 44.7 percent (21), and other trauma accounts for 40.4 percent (19). Vascular disease, congenital issues, and other causes make up the remainder.

* Type of assistive device: 85.1 percent (40) use a Prosthesis and 14.9 percent (7) use an Orthosis.

* P C - P T S D - 5 (at pre 1): 21 participants reported a traumatic event. 11 participants scored above the threshold of 3 or more, while 10 scored below the threshold of less than 3.

Navigate back to Table 1..

### Figure 1. Long description

The figure consists of four panels labeled by outcome. Each graph plots Score on the y-axis against Time on the x-axis with three intervals: Pre 1, Pre 2, and Post. Data points include error bars representing standard error.

* Top-left panel, Outcome K 10 underscore nervous: The score starts at approximately 4.1 at Pre 1, remains stable at 4.15 at Pre 2, then drops sharply to approximately 2.5 at Post.

* Top-right panel, Outcome K 10 underscore agitation: The score begins at 4.15 at Pre 1, decreases slightly to 4.0 at Pre 2, and then drops sharply to approximately 2.45 at Post.

* Bottom-left panel, Outcome K 10 underscore fatigue: The score starts at 4.45 at Pre 1, decreases to 4.2 at Pre 2, and drops sharply to approximately 2.7 at Post.

* Bottom-right panel, Outcome K 10 underscore negative A: This graph uses a different y-axis scale from 5 to 9. The score shows a linear decrease from approximately 8.9 at Pre 1, to 7.5 at Pre 2, and finally to 5.6 at Post.

Navigate back to Figure 1..

### Table 2. Long description

The table consists of four columns and five data rows.

Row 1: Quantitative domain K 10 is Nervousness. Quantitative result shows a significant reduction post-intervention. Qualitative theme is Theme 2: Peer support as emotional relief and encouragement. Illustrative interpretation states that sharing experiences and receiving support may have contributed to reduced anxiety and emotional tension.

Row 2: Quantitative domain K 10 is Agitation. Quantitative result shows a significant reduction post-intervention. Qualitative themes are Theme 2: Peer support as emotional relief and encouragement and Theme 3: Psychological and behavioural shifts. Illustrative interpretation states participants described feeling calmer and better able to manage emotional reactions.

Row 3: Quantitative domain K 10 is Negative affect. Quantitative result shows a significant reduction post-intervention. Qualitative theme is Theme 3: Psychological and behavioural shifts. Illustrative interpretation states improved emotional regulation and fewer interpersonal difficulties may help explain reductions in negative affect.

Row 4: Quantitative domain K 10 is Fatigue. Quantitative result shows a significant reduction post-intervention. Qualitative theme is Theme 1: Reclaiming social value and familial Role. Illustrative interpretation states re-engagement in meaningful family and community roles appeared linked to greater purpose and motivation.

Row 5: Quantitative domain K 10 is Not captured in K 10. Quantitative result is a dash. Qualitative themes are Theme 1, 2 and 3 (cross-cutting). Illustrative interpretation states social connectedness, hope, empowerment and relational belonging were central to participants experiences but not directly measured.

Navigate back to Table 2..
