# Supplementary material for: Mental health outcomes of a peer support programme for prosthesis and orthosis users in Cambodia: An explanatory sequential mixed-methods study
Source: Glob Ment Health (Camb). 2026 Jul 20;13:e163. doi: 10.1017/gmh.2026.10276 (PMC13433334; doi:10.1017/gmh.2026.10276)
Supplement: Supplementary file 1 [file S2054425126102763sup001.docx]

*Supplementary table 1- Confirmatory Factor Analysis (CFA) results based on previously published factor models*

| **Model** | **χ² (df)** | **CFI** | **TLI** | **RMSEA** | **SRMR** | **Loadings** |
| --- | --- | --- | --- | --- | --- | --- |
| **One-Factor** | 421.18 (35) | 0.83 | 0.79 | 0.154 | 0.068 | 0.595–0.838 |
| **Two-Factor (Brooks et al., 2006)** | 346.71 (34) | 0.87 | 0.83 | 0.141 | 0.055 | 0.61–0.84 |
| **Four-Factor (Brooks et al., 2006)** | 205.31  (29) | 0.924 | 0.882 | 0.114 | 0.038 | 0.58–0.82 |
| **Four-Factor (Hoffman et al., 2022)** | 242.44 (29) | 0.908 | 0.87 | 0.126 | 0.050 | 0.58–0.82 |

*Abbreviations: χ² = chi-square statistic; df = degrees of freedom; CFI = Comparative Fit Index; TLI = Tucker–Lewis Index; RMSEA = Root Mean Square Error of Approximation; SRMR = Standardized Root Mean Square Residual*.

*Supplementary table 2 – LMM results for the K10 Nervous domain*

| **Fixed effects** | | | | | | |
| --- | --- | --- | --- | --- | --- | --- |
| **Predictor** | **Estimate (β)** | | **SE** | **df** | **t** | **p** |
| Intercept | 2.21 | | 0.34 | 98 | 6.43 | <.001*** |
| Time |  | |  |  |  |  |
| – Pre1 | 1.43 | | 0.23 | 98 | 6.32 | <.001*** |
| – Pre2 | 1.52 | | 0.24 | 98 | 6.28 | <.001*** |
| Covariates |  | |  |  |  |  |
| Age <55 | 0.34 | | 0.19 | 98 | 1.76 | .082 |
| Sex (female vs. male) | -0.27 | | 0.28 | 98 | -0.98 | .328 |
| Urban vs. rural | 0.23 | | 0.23 | 98 | 0.99 | .323 |
| Disability – other | 0.07 | | 0.19 | 98 | 0.37 | .714 |
| Trauma exposure (yes) | 0.19 | | 0.19 | 98 | 0.98 | .329 |
| Education level 2 | 0.06 | | 0.22 | 98 | 0.29 | .776 |
| Education level 3 | 0.36 | | 0.28 | 98 | 1.32 | .189 |
| **Random effects** | | | | | | |
|  | Variance | | | | SD | |
| Participant (intercept) | 0.00 | | | | 0.00 | |
| Residual variance | 0.64 | | | | 0.80 | |
| **Model fit** | | | | | | |
| AIC | | BIC | | | | |
| 254.9 | | 291.1 | | | | |

*Supplementary table 3 – LMM results for the K10 Agitation domain*

| **Fixed effects** | | | | | | |
| --- | --- | --- | --- | --- | --- | --- |
| **Predictor** | **Estimate (β)** | | **SE** | **df** | **t** | **p** |
| ***Intercept*** | *2.25* | | *0.27* | *98* | *8.24* | *<.001**** |
| ***Time*** |  | |  |  |  |  |
| *– Pre1* | *1.70* | | *0.18* | *98* | *9.48* | *<.001**** |
| *– Pre2* | *1.68* | | *0.19* | *98* | *8.73* | *<.001**** |
| ***Covariates*** |  | |  |  |  |  |
| *Age <55* | *-0.02* | | *0.15* | *98* | *-0.16* | *.872* |
| *Sex (female vs. male)* | *0.25* | | *0.22* | *98* | *1.13* | *.263* |
| *Urban vs. rural* | *-0.11* | | *0.18* | *98* | *-0.63* | *.532* |
| *Disability – other* | *-0.11* | | *0.15* | *98* | *-0.73* | *.466* |
| *Trauma exposure (yes)* | *0.37* | | *0.15* | *98* | *2.43* | *.017** |
| *Education level 2* | *0.30* | | *0.17* | *98* | *1.73* | *.088* |
| *Education level 3* | *-0.05* | | *0.22* | *98* | *-0.24* | *.808* |
| **Random effects** | | | | | | |
|  | Variance | | | | SD | |
| Participant (intercept) | 0.00 | | | | 0.00 | |
| Residual variance | 0.404 | | | | 0.636 | |
| **Model fit** | | | | | | |
| AIC | | BIC | | | | |
| 215.3 | | 248.9 | | | | |

*Supplementary Table 4 – LMM results for the K10 Fatigue domain*

| **Fixed effects** | | | | | |
| --- | --- | --- | --- | --- | --- |
| **Predictor** | **Estimate (β)** | **SE** | **df** | **t** | **p** |
| **Intercept** | 2.58 | 0.31 | 43.08 | 8.33 | <.001*** |
| **Time** |  |  |  |  |  |
| – Pre1 | 1.62 | 0.17 | 66.12 | 9.74 | <.001*** |
| – Pre2 | 1.71 | 0.18 | 61.61 | 9.66 | <.001*** |
| **Covariates** |  |  |  |  |  |
| Age <55 | 0.19 | 0.17 | 42.11 | 1.10 | .280 |
| Sex (female vs. male) | 0.32 | 0.25 | 37.83 | 1.29 | .205 |
| Urban vs. rural | -0.37 | 0.21 | 38.14 | -1.79 | .082 |
| Disability – other | -0.31 | 0.17 | 34.35 | -1.78 | .084 |
| Trauma exposure (yes) | 0.93 | 0.15 | 96.43 | 6.09 | <.001*** |
| Education level 2 | 0.22 | 0.20 | 38.90 | 1.12 | .271 |
| Education level 3 | 0.09 | 0.2 |  |  |  |
| **Random effects** | | | | | |
|  | Variance | | | SD | |
| Participant (intercept) | 0.081 | | | 0.284 | |
| Residual variance | 0.322 | | | 0.568 | |
| **Model fit** | | | | | |
| AIC | BIC | | | | |
| 212.1 | 245.7 | | | | |

*Supplementary table 5 – LMM results for the K10 Negative affect domain*

| **Fixed effects** | | | | | |
| --- | --- | --- | --- | --- | --- |
| **Predictor** | **Estimate (β)** | **SE** | **df** | **t** | **p** |
| **Intercept** | 2.835 | 1.246 | 98 | 2.275 | 0.025* |
| **Time** |  |  |  |  |  |
| – Pre1 | 3.448 | 0.820 | 98 | 4.205 | <0.001** |
| – Pre2 | 2.784 | 0.878 | 98 | 3.171 | 0.002** |
| **Covariates** |  |  |  |  |  |
| Age <55 | -0.358 | 0.701 | 98 | -0.511 | 0.611 |
| Sex (female vs. male) | 1.975 | 1.007 | 98 | 1.963 | 0.053. |
| Urban vs. rural | 0.331 | 0.835 | 98 | 0.396 | 0.693 |
| Disability – other | 0.429 | 0.695 | 98 | 0.617 | 0.539 |
| Trauma exposure (yes) | 3.066 | 0.689 | 98 | 4.447 | <0.001** |
| Education level 2 | 0.752 | 0.797 | 98 | 0.945 | 0.347 |
| Education level 3 | 0.849 | 1.002 | 98 | 0.847 | 0.399 |
| education_level 4 | 1.840 | 1.241 | 98 | 1.482 | 0.141 |
| **Random effects** | | | | | |
|  | Variance | | | SD | |
| Participant (intercept) | 0.00 | | | 0.00 | |
| Residual variance | 8.39 | | | 2.89 | |
| **Model fit** | | | | | |
| AIC | BIC | | | | |
| 512.6 | 546.2 | | | | |
