# Supplementary material for: Mental health outcomes of a peer support programme for prosthesis and orthosis users in Cambodia: An explanatory sequential mixed-methods study
Source: Glob Ment Health (Camb). 2026 Jul 20;13:e163. doi: 10.1017/gmh.2026.10276 (PMC13433334; doi:10.1017/gmh.2026.10276)
Supplement: Peer review history

# Reviewer report: Review: Mental health outcomes of a peer support programme for prosthesis and orthosis users in Cambodia: An explanatory sequential mixed-methods study — R0/PR2

**DOI:** 10.1017/gmh.2026.10276.pr2

Overall, this was an interesting and well done studies. Below are several comments about improving the clarity of your manuscript.

Title:

- don’t capitalize “impact” and capitalize “An” after the colon.

- Is it a mental health program, or the impact of peer support on mental health Seems to be the later.

- Use “program” in the title and “programme” throughout

The impact statement is very powerful.

Abstract:

- Lines 5 – 6. Please provide more clarity on the 27 participants who took part in the intervention and the 20 people who completed baseline surveys. I think it would be more helpful to first introduce what kinds of data collection and then how many people completed each type. For example, how many people completed the K10 measure at all 3 time points?

Introduction:

- Provides a good overview. Some mechanical things to consider below.

- Page 2, line 1: what do you mean by “resource-sensitive”?

- Page 2 line 3: what are the “Sustainable Development Goals”? Why are they capitalized? Is it from a specific organization?

- Page 2, line 5: sometimes use LMIC and sometime spell it out. Be consistent.

- Page 2, line 10: What is the proportion of people with disabilities per capita according to Gartrell & Hoban?

- Page 2, line 17: Can you provide a comparison of how many psychiatrists they are in other countries for context?

- Page 3, line 8: I don’t understand why it says “, peer support,”. This sentence is unclear.

- Page 3, line 11: Please provide more details about this research. Where was it completed? Also, research cannot “develop and pilot” something. The grammar of this sentence is incorrect.

Methods:

- Note: sometimes friendship group is capitalized and sometimes it is not.

- Page 3, line 23-25: make it clear that the quantitative phase is completed before and after the intervention.

- Page 4, line 7: location should be plural.

- Page 4, line 10: did they have to use some kind of prosthetic or orthotic device?

- Page 4, line 14: you already say that Kessler Psychological Distress Scale = K10

- Page 4, line 16 (12 – 23): was completing the “friendship group” an inclusion criteria? Who was invited to complete the friendship group? Did people complete the friendship group (and score between 20 – 29), but not be invited to the study? When was the other K10 tests administered (in relation to the qualitative interviews)? This section leaves me with a lot of questions about what actually happened and when.

- Page 5, line 4: “It consists of an 8 week program that includes behavioural activation (explain what that means), structured peer discussion, and simple psychoeducational exercises (explain what that means)”

- Page 5, lines 21 – 23. Where there examples of what was urban vs rural? Did they collect information on type of prosthesis or orthosis (i.e. upper vs lower extremity, proximal vs distal amputations?)?

- Page 6, line 12 – 15: what determines it has “good” psychometric properties?

- Page 6, line 23: which approach did you go with? Brooks or Hoffman? Say you will evaluate it yourself.

- Page 7, line 4: who did the translation of the focus groups? How did you ensure that vital information was not lost in translation?

- Page 7, line 12: but above you said “The instrument has been extensively tested for validity, reliability and has been shown to have good psychometric properties as well as good cross-cultural validity…”. This is contradicting.

- Page 8, line 3: why did you choose landmine vs other?

- Page 8, line 4: how is education continuous? You said above there were 5 options?

- Page 8, line 14: “Friendship Group” is capitalized here, not elsewhere.

- Page 8, line 25: you talk about coding and then making themes and then back to coding. I would keep it linear, unless you checked the coding after you had developed themes?

- Page 9, line 3: how do you focus on participant’s language when it is translated? Do you lose anything in that?

- Overall, can you provide more information on who the interviewers were and their experience, how the interviews were translated and what was prioritized (it being word for word? Overall concept? Etc?) Were translators and interviewers included in the analysis and theme creation? Were they invited to be reflexive? Were Cambodians included in the analysis and result generation?

Table 1:

- What are the numbers in the brackets? I’m assuming they are percents? But one says 106. Please confirm and make clear.

- Can you present age as a continuous variable with mean, standard deviation and range?

- Given the numbers are small, would it make sense to combine divorced and widowed with single?

- How does housework relate to income?

- I don’t understand the orthotic categories. Why split out the one person who had a congenital impairment and an orthosis? What is “other lower limb impairment” vs “other”?

- Should # exposed to traumatic event equal those below + above the threshold? Because right now they don’t add up and it’s confusing.

- You are missing brackets on the CI for the Pretest 1.

Results

- Page 9, line 11: what other inclusion criteria did they meet besides scores?

- Page 9, line 12: Were all 47 invited to do the friendship group?

- Page 12, line 7: you described above the cross-loading in the model and then say it was the best factor. Acknowledge the limitations of using this model.

- Pages 12 – 14: the results on each of the domain scores are very repetitive. Please combine them for more succinct results.

- Page 14, line 10-11. Based on figure 1, it appears that negative effect is significantly lower and test2 than test1.

- Page 15, line 24-25: the second half of this quote is not grammatically correct. While it might be what the participant said, I would reword for increased readability. This idea comes up a lot. Given that the interviews were translated, I think your quotes need to be worded much better. Right now, the reader wastes their energy trying to decipher what they are saying. It also shows your participants in a better light. This is true for almost all of the quotes.

- Page 16, line 1: This quote just lists things but doesn’t add anything to the results. Be selective in the quotes you chose and how they add to your results.

- Theme 1: do you have any quotes that speak specifically to social value? You list the things that people do, but not how it gives them value. I think this theme could be strengthened by going a step beyond showing what they do to demonstrating how they feel.

- Page 16, line 11 – 19: just use the second quote. Or put some of your commentary between it. Don’t just list quotes back to back. This applies in several other places as well.

- Overall, I think that this section needs to be edited for better flow. Select only the most important quotes and edit them for grammar. Insert your own commentary and description between the quotes. If you do this, you will have a strong results section.

Discussion

- Page 18, line 24: is this statistically lower, clinically relevant?

- Page 20, line 4: “likely” based on what?

- Page 20, line 5-6: “Rather than reflecting physical recovery, the quantitative drop in fatigue seems to represent renewed psychological vitality.” What is this claim based on?

- Overall, the discussion includes many helpful comparison to other literature, but does so while reiterating the results several times. Try to reduce result reiteration and focus on interpreting and comparing.

- Page 21, line 7: “which allowed statistical…”?

- Page 21, line 21: I think translation is a much more important limitation. Especially considering the grammar in the quotes presented.

Conclusion:

- Page 22, line 1: sometimes mixed methods and sometimes mixed-methods.

- Page 22, line 3: the program does not show significant reductions, the people who attend do

Supplemental table

- Table 1: include the abbreviations

# Reviewer report: Review: Mental health outcomes of a peer support programme for prosthesis and orthosis users in Cambodia: An explanatory sequential mixed-methods study — R0/PR3

**DOI:** 10.1017/gmh.2026.10276.pr3

**Authors:** Bernd Puschner

**Affiliations:** Department of Psychiatry II, Ulm University, Germany

This paper reports results of a mixed-methods study about a group peer support intervention to improve mental health of people with physical disabilities in Cambodia. It is timely and well-written, but also has some problems.

1 Title, Abstract, Impact Statement

(a) The use of the term “impact” in the title implies that the study demonstrates a causal effect of the intervention. However, given the pre–post design without a comparator group, the study does not permit a robust assessment of effectiveness. This is an important limitation that is not adequately acknowledged in the manuscript. It is only briefly noted in the conclusion (“While this was not a randomised trial…”) and is not sufficiently discussed in the limitations section. While a randomised controlled trial may not always be feasible or necessary, the authors should adopt a more critical perspective on the implications of their design. It may be more appropriate to frame the study as exploratory or as a pilot investigation, with greater emphasis on theory development through an integrated synthesis of the quantitative and qualitative findings (cf. 4c).

(b) Relatedly, the limitations inherent to this design should be explicitly and consistently addressed throughout the manuscript. Statements that imply effectiveness has been established should be revised. This includes more subtle instances, such as in the abstract, where the statement “27 participants took part in the intervention while 20 completed baseline surveys only” may inadvertently suggest the presence of a control group, which is not the case. Greater precision in wording would help avoid misinterpretation.

(c) Please explain what is meant by “In a context where mental health remains highly sensitive …”, or rephrase (probably related to high stigma).

2 Introduction

(a) I have reservations about positioning peer support within a task-shifting framework, as this risks overlooking its distinct nature as a recovery-oriented intervention grounded in lived experience. Framing peer support in this way may inadvertently reduce it to a substitute for professional roles, rather than recognising it as a complementary and unique form of expertise. Similarly, statements such as “under appropriate supervision from mental health specialists” suggest that individuals with lived experience are not regarded on equal footing with other mental health professionals. Instead, they appear to be framed as “individuals without formal qualifications” who are delegated “tasks traditionally performed by trained professionals”. This reflects an insufficient consideration of the perspective and contributions of people with lived experience. I would therefore encourage a more detailed description of their role within the project—not only as service providers, but also in the planning, design, and implementation of the programme as a whole. If their involvement in these areas has been confined, this should be explicitly acknowledged as a limitation.

(b) Furthermore, I found the relationship between this study and a previous, very similar study [1] insufficiently clarified. Given the authors’ own statement that this work was “in preparation for a larger pilot RCT including a control group,” a logical next step would have been a design capable of establishing effectiveness. The manuscript would benefit from a clearer explanation of how the two studies relate to one another, how the current study builds upon the previous work, and why this anticipated next step was not undertaken.

3 Methods

(a) “Physical disabilities” is listed as an inclusion criterion. However, then only people using prostheses and orthoses are included. I imagine there are many physical disabilities not requiring such devices. Please explain.

(b) If the “validated Khmer version of the K10” has been used, then why making such a big effort to investigate its construct validity? At the same time, the total score is used in many instances (baseline and first pre-test), despite its flawed construct validity, and then not any more in the pre-post tests which exclusively focussed on findings on the four domains (without knowing which of the 10 item belong to which factor). Given the overall aim of this paper, devoting so much attention to psychometrics appears questionable, even more as it is still incomplete as other important psychometric properties, e.g. test-retest reliability, criterion validity, sensitivity to change, and internal consistency, are not reported. I suggest to reconsider whether this is necessary at all, and to avoid inconsistencies in the use of total vs. domain scores throughout the paper. Maybe using the total score throughout would be best, also because the four factors are made up of very few items, mostly only two.

(c) When reporting results, you might consider also tracking the proportion of participants shifting between distress categories (e.g. from “severe” to “moderate”) as this is often more interpretable than mean score differences alone.

(d) While the K-10 is an established measure of distress, it does not target specific effects of peer support, such as social connectedness, hope and empowerment. I acknowledge that these aspects have been taken into account in the qualitative part of this paper. Nonetheless, not using a recovery-oriented measure as a secondary outcome in the quantitative part is a caveat which should be acknowledged.

(e) Please add information about the recall window used for the K10, as this might affect outcome assessment, especially if very long (30+ days).

(f) Please include the complete topic guide used in the focus groups as a supplement.

4 Results

(a) Post-test data are reported in the last row of Table 1 in the “Pretest data only” column. Please check.

(b) “Not all participants knew their exact age”: How could age then be reported for all participants in Table 1? Please clarify.

(c) Synthesis of results from quantitative and qualitative strands of this study is intertwined unsystematically in the discussion section. However, it is recommended to provide an adequate meaningful integration of qualitative and quantitative data from this convergent mixed methods design as last part of the results section, by using appropriate methods, e.g. a joint display (matrix or figure) [2].

(d) It would also be worthwhile to show the complete coding tree.

5 Discussion

(a) The discussion should have a clearer focus and structure. Please add subheadings, shorten, and omit results of the integration of qualitative and quantitative data, to be part of the results section (see 4c).

(b) Please also make an effort to refrain from speculations (“may have been unevenly felt” etc.).

References

1. Best P, Maddock A, Ean N, Montgomery L, Armour C, Mulholland C, Blair C. Developing and testing a community based, online vs. face-to-face peer led intervention to improve mental well-being in Cambodian adults with physical disabilities. Frontiers in Digital Health. 2024;Volume 6 - 2024.

2. Guetterman TC, Fetters MD, Creswell JW. Integrating Quantitative and Qualitative Results in Health Science Mixed Methods Research Through Joint Displays. Ann Fam Med. 2015;13:554–61. doi:10.1370/afm.1865.

# Editor report: Recommendation: Mental health outcomes of a peer support programme for prosthesis and orthosis users in Cambodia: An explanatory sequential mixed-methods study — R0/PR4

**DOI:** 10.1017/gmh.2026.10276.pr4

**Affiliations:** Alcohol Tobacco and Other Drug Research Unit, South African Medical Research Council, South Africa

We are pleased to now give you feedback from two peer reviewers. We request that you particularly take into consider Reviewer 2 comments around the framing of the work as more exploratory, instead of showing impact/effectiveness.

# Editor report: Decision: Mental health outcomes of a peer support programme for prosthesis and orthosis users in Cambodia: An explanatory sequential mixed-methods study — R0/PR5

**DOI:** 10.1017/gmh.2026.10276.pr5

**Affiliations:** Johns Hopkins University Bloomberg School of Public Health, United States

No accompanying comment.

# Reviewer report: Review: Mental health outcomes of a peer support programme for prosthesis and orthosis users in Cambodia: An explanatory sequential mixed-methods study — R1/PR7

**DOI:** 10.1017/gmh.2026.10276.pr7

**Authors:** Bernd Puschner

**Affiliations:** Department of Psychiatry II, Ulm University, Germany

The authors have adequately taken into account my comments in the revision, the paper has improved a lot. I only have one remaing concern. I believe that my comment

“I would therefore encourage a more detailed description of their role within the project—not only as service providers, but also in the planning, design, and implementation of the programme as a whole. If their involvement in these areas has been confined, this should be explicitly acknowledged as a limitation.”

has not been considered enough. Thus, I again ask the authors to decribe in detail how the expertise of people with lived experience has been taken into account in the different domains of the project, and if this has not happened, to clearly mention this as a limitation.

# Reviewer report: Review: Mental health outcomes of a peer support programme for prosthesis and orthosis users in Cambodia: An explanatory sequential mixed-methods study — R1/PR8

**DOI:** 10.1017/gmh.2026.10276.pr8

The authors did a great job of responding to the comments. I have a few more things to improve readability.

Impact statement: still says “programme” throughout. Otherwise, the changes look great.

Abstract: this is much improved. The only thing I’d add is that it says twice that non-participants contributed baseline data only. It is a bit repetitive but it is clear.

Page 2, line 8: Nations’

Page 2, line 16: I think it would help strengthen your paper to build out the evidence behind this number a little bit. 9% seems low. For example, according to statistics Canada, ~27% of adults have a disability in Canada. If Cambodia has the highest proportion per capita, I’d expect it to be higher than 9%. If this discrepancy is due to measurement or categorization differences, perhaps state that? Could also be helpful to emphasize that disability may be more limiting in this context with more physical tasks required for daily living and less financial support available?

Page 3, line 23: I would add something to the first sentence about the aim being to evaluate psychological distress for those who completed the program. Being a bit more specific in articulating your aim would be helpful. On the next page, it states the study was used to “explore changes in mental health outcomes following participation in an 8 week long friendship group intervention”. This is more clear than the aim statement above.

Table 1 looks good. I don’t think you need to report infection and cancer if there are none. Also for “average income” I’d specify the time period (e.g., weekly, monthly, yearly…)

The themes read better with more explanation between the quotes.

Page 13, Line 6: Is it “time” or the “intervention”?

Page 15 line 14 & Page 17, Line 23: the same quote is included twice. I would try to find a different quote or in the second place, mention the one above instead of restating it.

I like the addition of table 2.

Limitations: this section seems very long. I would condense it a bit and edit it for better flow between paragraphs. The first paragraph does not transition into the other ones very well – I thought it was an introduction to the limitations you were discussing more below, but this is not the case.

Overall, excellent job addressing the prior feedback.

# Editor report: Recommendation: Mental health outcomes of a peer support programme for prosthesis and orthosis users in Cambodia: An explanatory sequential mixed-methods study — R1/PR9

**DOI:** 10.1017/gmh.2026.10276.pr9

**Affiliations:** Alcohol Tobacco and Other Drug Research Unit, South African Medical Research Council, South Africa

Please take into consideration the final comment of Reviewer 1.

# Editor report: Decision: Mental health outcomes of a peer support programme for prosthesis and orthosis users in Cambodia: An explanatory sequential mixed-methods study — R1/PR10

**DOI:** 10.1017/gmh.2026.10276.pr10

**Affiliations:** Johns Hopkins University Bloomberg School of Public Health, United States

No accompanying comment.

# Editor report: Recommendation: Mental health outcomes of a peer support programme for prosthesis and orthosis users in Cambodia: An explanatory sequential mixed-methods study — R2/PR12

**DOI:** 10.1017/gmh.2026.10276.pr12

**Affiliations:** Alcohol Tobacco and Other Drug Research Unit, South African Medical Research Council, South Africa

Thank you for addressing the reviewers final comments.

# Editor report: Decision: Mental health outcomes of a peer support programme for prosthesis and orthosis users in Cambodia: An explanatory sequential mixed-methods study — R2/PR13

**DOI:** 10.1017/gmh.2026.10276.pr13

**Affiliations:** Johns Hopkins University Bloomberg School of Public Health, United States

No accompanying comment.
